# Caregivers’ compliance with referral advice: evidence from two studies introducing mRDTs into community case management of malaria in Uganda

**DOI:** 10.1186/s12913-018-3124-8

**Published:** 2018-05-02

**Authors:** Sham Lal, Richard Ndyomugenyi, Lucy Paintain, Neal D. Alexander, Kristian S. Hansen, Pascal Magnussen, Daniel Chandramohan, Siân E. Clarke

**Affiliations:** 1 Department of Disease Control, Faculty of Infectious Tropical Diseases, London School of Hygiene and Tropical Medicine, Keppel Street, London, UK; 2 C/O Vector Control Division, Ministry of Health, Kampala, Uganda; 3 MRC Tropical Epidemiology Group, Department of Infectious Disease Epidemiology, Faculty of Epidemiology and Population Health, London School of Hygiene and Tropical Medicine, London, UK; 4 Section of Health Services Research, Department of Public Health, University of Copenhagen, Copenhagen, Denmark; 5 Department of Immunology and Microbiology, Centre for Medical Parasitology, University of Copenhagen, Copenhagen, Denmark; 6 Department of Veterinary Disease Biology, Faculty of Health and Medical Sciences, University of Copenhagen, Copenhagen, Denmark

**Keywords:** Community health workers, Malaria, Referral, Compliance, Uganda, Epidemiology, Under-5

## Abstract

**Background:**

Several malaria endemic countries have implemented community health worker (CHW) programmes to increase access to populations underserved by health care. There is considerable evidence on CHW adherence to case management guidelines, however, there is limited evidence on the compliance to referral advice and the outcomes of children under-5 referred by CHWs. This analysis examined whether caregivers complied with CHWs referral advice.

**Methods:**

Data from two cluster (village) randomised trials, one in a moderate-to-high malaria transmission setting, another in a low-transmission setting conducted between January 2010–July 2011 were analysed. CHW were trained to recognise signs and symptoms that required referral to a health centre. CHW in the intervention arm also had training on; malaria rapid diagnostic tests (mRDT) and administering artemisinin based combination therapy (ACT); CHW in the control arm were trained to treat malaria with ACTs based on fever symptoms. Caregivers’ referral forms were linked with CHW treatment forms to determine whether caregivers complied with the referral advice. Factors associated with compliance were examined with logistic regression.

**Results:**

CHW saw 18,497 child visits in the moderate-to-high transmission setting and referred 15.2% (2815/18,497) of all visits; in the low-transmission setting, 35.0% (1135/3223) of all visits were referred. Compliance to referral was low, in both settings < 10% of caregivers complied with referral advice. In the moderate-to-high transmission setting compliance was higher if children were tested with mRDT compared to children who were not tested with mRDT. In both settings, nearly all children treated with pre-referral rectal artesunate failed to comply with referral and compliance was independently associated with factors such as health centre distance and day of referral by a CHW. In the moderate-to-high transmission setting, time of presentation, severity of referral were also associated with compliance, whilst in the low-transmission setting, compliance was low if an ACT was prescribed.

**Conclusions:**

This analysis suggests there are several barriers to comply with CHWs referral advice by caregivers. This is concerning for children who received rectal artesunate. As CHW programmes continue scale-up, barriers to referral compliance need to be addressed to ensure a continuum of care from the community to the health centre.

**Trial registration:**

The study was registered with ClinicalTrials.gov. Identifier NCT01048801, 13th January 2010.

**Electronic supplementary material:**

The online version of this article (10.1186/s12913-018-3124-8) contains supplementary material, which is available to authorized users.

## Background

Globally there have been considerable declines in malaria mortality rates during the past decade, but progress has been slowest in countries where the burden of malaria is highest and where access to primary healthcare is most limited [1]. In Uganda, where malaria accounts for approximately 18% of all deaths in children under-5 years, approximately 65% of the population lived more than 5 km from the nearest government health centre [2, 3]. To address the disease burden and increase access to healthcare, community health worker (CHW) programmes such as integrated community case management (iCCM), have been extensively supported by WHO, UNICEF and The Global Fund since 2012 [4]. Studies suggest that when CHW are appropriately trained, supplied and supported, they can increase access to healthcare and reduce under-5 mortality by providing primary healthcare closer to homes of children at risk of malaria [5–7].

A CHW is typically a member of the community with little or no previous professional medical experience, but are trained to diagnose and treat a small number of specific diseases, often including malaria [8]. A crucial component of CHW training programmes is to identify and refer children who require the attention of higher-level healthcare professionals who are better equipped and trained to manage a wider range of clinical conditions [9]. For a community based referral system to function optimally, the CHW should first be able to identify children requiring referral based on signs and symptoms and to advise caregivers to take the child to a referral centre; second the caregivers should comply with CHWs referral advice and seek care from health centres; and third health centres should be equipped and ready to manage appropriately the referred children [10]. Progression through each of these stages is essential to help avoid treatment delays and possibly death.

iCCM has become national healthcare policy in 33 sub-Saharan African (SSA) countries, informed by an ever growing evidence base on how to implement and scale-up this approach. In comparison, data on the effectiveness of referral systems remains limited, despite being an integral component of primary healthcare [11, 12]. Prior studies have usually reported low compliance with referral advice for example, studies of CHW using mRDTs for only malaria case management in Sierra Leone and Zambia found that 98% and 70% of caregivers respectively, did not comply with referral advice [13, 14]. Poor compliance has also been reported in recent iCCM programmes, with less than 46% of all caregivers complying with referral [15, 16]. Yet relatively few studies have examined the barriers that hinder caregivers’ compliance with referral advice. The limited evidence base on referral has been highlighted as a priority research area by the international task force on iCCM [17]. In this analysis, we explored caregivers’ compliance to CHW referral advice in relation to the demographic, geographical and temporal barriers that might affect compliance, using data collected during two cluster randomised trials to introduce malaria rapid diagnostic tests (mRDT) in community case management in rural Uganda.

## Methods

### Study area and participants

The two trials were conducted in two sub-counties in Rukungiri District, South-west Uganda; one trial in a moderate-to-high malaria transmission setting (Bwambara sub-county, 980 m–1200 m above sea level) and the other in an epidemic-prone low transmission setting (Nyakishenyi sub-county, 1064 m–2157 m above sea level). In each setting, approximately 20% of the population were aged under 5 years [18]. Both settings were characterised by hilly terrain, where 86% of the population lived in rural areas, the predominant livelihood was subsistence farming, and walking was often the main form of transport [19, 20]. The public health system in each setting was comprised of three government health centres: two classed as Health Centre II (HCII) and one classed as Health Centre III (HCIII). HCIIs are typically staffed by two enrolled nurses providing basic outpatient care and community outreach services, while HCIIIs also have the capacity to admit patients and supervise the lower level HCIIs. Both HCIIs and HCIIIs serve as a referral point for CHW [21].

Detailed information on the design and procedures of each trial is available elsewhere [22–24]. In brief, within each sub-country, CHWs were randomised to either a current practice or intervention arm; the latter receiving training in mRDT-based diagnosis of malaria. In all other respects, the training received by CHWs was identical in both arms. This training included how to treat uncomplicated malaria in children and how to recognise symptoms of other febrile illnesses that should be referred to the nearest health centre. All 381 CHWs (192 in the moderate-to-high transmission setting, 189 in the low-transmission setting) were thus trained to identify severe and non-severe signs and symptoms for referral in children presenting with fever (Table 1). Severe signs and symptoms requiring urgent referral included convulsions or fits, extreme weakness, coma/loss of consciousness and very hot body temperature of 38.5 °C; these symptoms were chosen to ensure children with indications compatible with meningitis, severe malaria, pneumonia, or severe bacterial infections were referred and managed at health centres as quickly as possible. Non-severe signs and symptoms included wounds, ear infections, sticky or red eyes, and vomiting and diarrhoea without signs of dehydration; selected to identify common and readily detectable conditions in children requiring management at health centres. These referral criteria were based on Uganda’s national treatment guidelines and the research team’s clinical experience [25].

**Table 1:** List of severe and non-severe signs and symptoms that community health workers (CHW) were trained to identify and refer in children

Severe sign and symptoms for urgent referral	Non-severe sign and symptoms for referral
Refer using emergency referral form if child shows any of the following symptoms:	Refer using ordinary referral form if child shows any of the following symptoms:
1. Illness in child below 2 months 2. Convulsions of fits now or within the past 2 days 3. Coma/loss of consciousness 4. Patient is confused or very sleepy-cannot be woken 5. Extreme weakness-unable to stand or sit without support 6. Very Hot-with temperature of 38.5 °C or more 7. Very Cold-with temperature of 35.0 °C of less 8. Vomiting everything-cannot keep down food or drink 9. Not able to drink of breast feed 10. Severe anaemia-very pale palms, fingernails, eyelids 11. Yellow eyes 12. Difficulty in breathing 13. Severe dehydration	1. Fever in babies less than 4 months old 2. Fever that has last for more than 7 days 3. Fever with measured temperatures of 37 °C or more and mRDT negative 4. Vomiting and diarrhea 5. Blood in faeces or blood in urine 6. Pain when passing urine or frequent urination 7. Wound or burns 8. Skin abscess 9. Painful swelling or lumps in the skin 10. Ear infection (runny ear or child pulling at the ear) 11. Sticky or red eyes
If RDT result is positive: Treat child (if older than 2 months) with rectal artesunate suppository prior to referral.	If RDT results is positive: Treat child (if older than 4 months) with artemether-lumefantine tablets prior to referral.

As well as the training on referral guidelines, CHWs in the intervention arm of each trial (93 in the moderate-to-high transmission setting, 96 in the low-transmission setting) were trained to diagnose malaria by using mRDTs and to only treat with antimalarials after a positive mRDT result. In contrast, control arm CHWs were trained to diagnose malaria based on a child’s presenting signs and symptoms. In both arms, all CHWs were trained to treat uncomplicated malaria with an oral age-dependent dose of an ACT (artemether-lumefantrine) and to administer pre-referral rectal artesunate when children presented with signs or symptoms of severe malaria and to refer them to the nearest health centre for further management. The job aids summarising the decisions CHW were trained to make in each arm are shown in Additional file 1: Figure S1 (further details of the trial and CHW training materials are available from: http://www.actconsortium.org/publications.php/83/training-manuals-use-of-artemisinin-based-combination-therapies-and-rapid-diagnostic-tests-for-home-.html

### Data collection

The trials in the moderate-to-high and low transmission settings began in May 2010 and June 2010 respectively. For the first 6 months of the trial (May/June 2010–December 2010) CHW were supervised frequently by field coordinators to discuss concerns or difficulties in carrying out their new roles. From January 2011 until trial completion in July 2012 supervision of CHWs was scaled back and limited to monthly group meetings to reflect operational programme conditions.

For every child that presented with a fever or a history of fever, the CHW recorded the history of illness including the child’s temperature, when the fever started, and whether the child had slept under a net the previous night, together with demographic details on name, age, gender, and village of residence, on a treatment recording form (TRF). Finally, CHWs recorded the mRDT result (intervention arm only), whether an ACT or rectal artesunate was prescribed and whether they had advised the caregiver to take the child to the nearest health facility. CHWs were asked to classify referrals as severe or non-severe referrals according to the symptoms and to complete either a severe referral form or a non-severe referral form accordingly. Both referral forms listed the referral signs and symptoms identified by the CHW; as well as the RDT test result (where applicable), and any malaria treatment given by the CHW. Caregivers were asked to report to health centres as soon as possible and a copy of the referral form was given to them to present at the health centre; with a duplicate carbon copy of the referral form kept by CHW for their records. At the health centre, health workers received the caregiver and their child and recorded their final diagnosis and treatment decisions on the referral form. Referral forms completed by the CHW and health centre staff were collected from the health centres on a regular basis by the research team.

To examine caregiver compliance to CHW referral advice, data were collected from the treatment recording forms, severe and non-severe referral forms over an 18-month period after the end of close support supervision (January 2011–July 2012). Caregiver’s compliance was defined as visiting a public health centre after being referred by a CHW. This was determined by record linkage between the CHW treatment record form, which captured whether a referral was made, and the referral form completed by health workers at health centres, both forms were linked in the database using the child’s unique identification number. The analysis examined factors found to influence referral compliance in previous studies [26, 27], such as child’s age, gender and the reported duration of fever. In addition, the analysis also examined whether the day of referral (weekday/weekend) and the season (wet/dry) were associated with differences in the caregiver’s referral compliance. These data were derived from the date of referral, and the season indicator variable was defined as the months that coincided with the two rainy seasons (March–May and September–December). The number of days taken to complete referral was also calculated from the date of referral and the date a caregiver visited the health centre. Finally, GPS coordinates were taken to measure Euclidean (straight-line) distance from the centre of a village to the nearest health centre.

### Statistical methods

All data were double entered and verified using Microsoft Access 2007 (Microsoft Inc., Redmond, Washington). Data were analysed using STATA version 14.1 (STATA Corporation, College Station, Texas). The outcome for this analysis was the proportion of caregivers that complied with CHW referral advice out of all caregivers who were referred by CHW. Since perceptions of the severity of malaria might differ between the two transmission areas, potentially influencing compliance with referral, data were analysed separately for the two sub-counties. Analysis of each trial dataset was analysed to examine whether caregiver compliance to CHW referral advice differed in relation to malaria testing with mRDTs or other factors (age, gender, net use, ACT prescribed, day of referral, season, distance to nearest health facility, severity of referral, time the since first onset of symptoms). For each child visit to a CHW, mRDT testing was defined as one of three mutually exclusive categories; (1) visits where CHW did not use a mRDT and diagnosis was presumptive; (2) visits where CHW used a mRDT and the mRDT result was positive; (3) visits where CHW tested with a mRDT and mRDT result was negative. For each trial, an explanatory model for the outcome of referral compliance was developed using logistic regression and odds ratios (OR) with 95% confidence intervals (95%CI) were calculated using random effects to account for clustering at the village level [28]. Factors identified *a priori* were included in the adjusted analysis and likelihood ratio tests were used.

## Results

### Study population

**Fig. 1:**
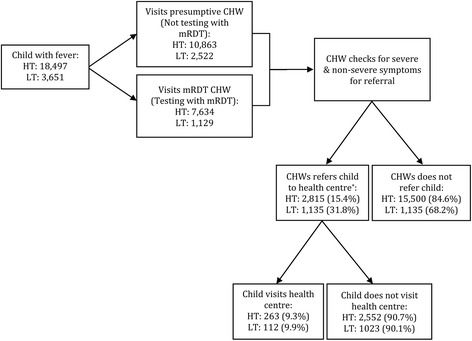
Flowchart of children analysed in the moderate-to-high malaria transmission setting (HT) and the low-transmission setting (LT). *Referral status missing for 182 child visits in the HT setting and 79 visits in the LT setting

Between January 2011 and July 2012, CHWs in the moderate-to-high transmission setting recorded 18,497 children with fever, of whom they referred 15.2% (2815/18,497) of all child visits (Fig. 1). Over the same period, CHWs in the low transmission setting saw fewer children but referred more frequently, with 35.0% (1135/3223) of all visits being referred (Fig. 1). The characteristics of referred children were broadly similar in both transmission settings, most were aged 1–3 years, approximately half were female, 80.0% or more had slept under a bed net the previous night, 83.5% lived in the same village as the CHW, 80.0% had visited a CHW within 24 h of fever onset however distance from the nearest health facility was greater for children referred in the low transmission setting (Table 2). In both settings, more referrals occurred on a weekday (70%) than during the weekend (30.0%), and around 60% of referrals were made in the wet season.

**Table 2:** Characteristics of children who were referred by CHWs

	Number of referrals by CHWs in the moderate-to-high transmission setting (%)^a^	Number of referrals by CHWs in the low-transmission setting (%)^b^
	*N* = 2815	*N* = 1135
Age (years)		
< 1.0	820 (29.4)	324 (29.0)
1.0–2.9	1169 (41.9)	483 (43.2)
3.0–4.9	788 (28.3)	302 (27.0)
5.0–15.0	10 (0.4)	9 (0.8)
Gender		
Male	1463 (52.4)	585 (51.8)
Female	1330 (47.6)	544 (48.2)
Slept under a net the previous night		
No	267 (9.6)	129 (11.6)
Yes	2500 (90.4)	984 (88.4)
Came from same village		
No	317 (11.3)	187 (16.5)
Yes	2490 (88.7)	945 (83.5)
Duration of fever (hours) since onset of symptoms		
> 24 h	408 (15.0)	220 (20.0)
Within 24 h	2321 (85.0)	879 (80.0)
Tested with mRDT		
Not tested	164 (5.8)	352 (31.0)
Tested	2651 (94.2)	783 (69.0)
Type of referral		
Severe referral signs	1013 (36.0)	421 (37.1)
Non-severe referral signs	1607 (57.1)	636 (56.0)
Day of referral		
Weekday	2022 (71.8)	791 (69.7)
Weekend	793 (28.2)	344 (30.3)
Season		
Dry	1139 (40.5)	419 (36.9)
Wet	1676 (59.5)	716 (63.1)
Village distance to nearest health facility (km)		
0.0–2.4	1578 (56.5)	296 (27.9)
2.5–4.9	1141 (40.9)	395 (37.2)
5.0–7.4	72 (2.6)	241 (22.7)
7.5–8.9	0 (0.0)	130 (12.2)

^a^Data missing on the number of referrals in the moderate-to-high transmission setting, for age: 28; sex: 22; net use: 48; resident in the same village: 8; onset of symptoms 86

^b^Data missing on the number of referrals in the low transmission setting, for age: 17; sex: 6; net use: 22; resident in the same village: 3; onset of symptoms 36

### Compliance to referral advice

Caregivers’ compliance to referral advice was low, with only 9.3% (263/2815) and 9.9% (112/1135) of referred children subsequently seen at a government health centre in the moderate-to-high transmission (Table 3) and low transmission (Table 4) settings respectively. The majority of caregivers in the low transmission setting who complied with referral did so on the same day as being referred by CHW (65.7% (69/105, 7 missing information)), but this was less frequent in the moderate-to-high transmission setting (49.4% (127/257, 6 missing information)). In both settings, caregivers who did not comply the same day, took between 2 to 10 days to visit a health centre. There were no differences in the days taken to complete referral in relation to whether a child was referred with severe or non-severe signs or symptoms (data not shown).

**Table 3:** Caretakers compliance to referral, in relation to mRDT results and ACT prescribing in moderate-to-high transmission setting

	Referrals by CHWs	Complied with referral (%)	Did not comply with referral (%)	*p*-value
Total	2815	263 (9.3)	2552 (90.7)	
mRDT result				
Not tested	164	7 (4.3)	157 (95.7)	
Tested	2651	256 (9.7)	2395 (90.3)	0.025
Within those tested with an mRDT				
mRDT negative	2558	244 (9.5)	2314 (90.5)	
mRDT positive	93	12 (12.9)	81 (87.1)	0.319
ACT prescription by CHW^a^				
ACT not prescribed	2538	242 (9.5)	2296 (90.5)	
ACT prescribed	82	8 (9.8)	74 (90.2)	0.961
Rectal artesunate prescribed	63	3 (4.8)	60 (95.2)	
Type of referral^b^				
Non-severe signs or symptoms	1607	111 (6.9)	1496 (93.1)	
Severe signs or symptoms	1013	152 (15.0)	861 (85.0)	< 0.001

^a^132 missing ACT prescription data

^b^195 missing type of referral

**Table 4:** Caretakers compliance to referral, in relation to mRDT results and ACT prescribing in low transmission setting

	Referrals by CHWs	Complied with referral (%)	Did not comply with referral (%)	p-value
Total	1135	112 (9.9)	1023 (90.1)	
mRDT result				
Not tested	352	34 (9.7)	318 (90.3)	
Tested	783	78 (10.0)	705 (90.0)	0.923
Within those tested with an mRDT				
mRDT negative	770	75 (9.7)	695 (90.3)	0.147
mRDT positive	13	3 (23.1)	10 (76.9)	
ACT prescription^a^				
ACT not prescribed	805	86 (10.7)	719 (89.3)	
ACT prescribed	218	10 (4.6)	208 (95.4)	0.031
Rectal artesunate prescribed	61	10 (16.4)	51 (83.6)	
Type of referral^b^				
Non-severe signs or symptoms	636	71 (11.2)	565 (88.8)	0.082
Severe signs or symptoms	421	41 (9.7)	380 (90.3)	

^a^51 missing ACT prescription data

^b^78 missing type of referral

The relationship between caregiver’s compliance and the diagnosis and treatment characteristics the child had received from the CHW (mRDT testing, ACT prescription and severity of illness) was also examined. In the moderate-to-high transmission setting, compliance was greater when children were tested for malaria using mRDTs compared children who were not tested (9.7% vs. 4.3%, *p* = 0.025) and more frequent amongst children with severe signs or symptoms compared with children with non-severe signs or symptoms (15.0% vs. 6.9%, *p* < 0.001, Table 3). However, there was no significant difference in compliance between mRDT positive or negative children, or whether a CHW prescribed an ACT. In contrast, in the low transmission setting there was no evidence to suggest compliance was associated with mRDTs or the severity of illness (Table 4). However, in this setting caregivers complied more frequently when an ACT was not prescribed compared to when it was prescribed (10.7% vs. 4.6%, *p* = 0.031, Table 4).

### Factors associated with compliance to referral advice

Tables 5 and 6 present a multivariable adjusted analyses of the factors associated with caregivers compliance to CHW referral advice in the two transmission settings respectively. In the moderate-to-high transmission setting, compliance with referral was less likely when children visited a CHW within 24 h of symptoms onset, compared to later (OR 0.50; 95% CI 0.37–0.69; *p* < 0.001). Compliance was also 37% less likely for children presenting with non-severe signs or symptoms for referral compared to children with severe signs and symptoms for referral (OR 0.63; 95% CI 0.44–0.90; *p* < 0.012) (Table 5). After controlling for other factors in the adjusted analyses, there was no association between referral compliance and the mRDT result, age or gender; however, it was found that compliance was less likely when children were referred during a weekend compared to a weekday and when referrals occurred during the wet season compared to the dry season. We also found compliance with referral advice declined with increasing distance from the nearest health centre in the moderate-to-high transmission setting (Table 5).

**Table 5:** Caregiver factors associated with referral compliance in the moderate-to-high transmission setting

Variables	Referrals by CHWs	Complied with referral (%)	Unadjusted odds Ratio (95% CI)	*p*-value	Adjusted odds ratio (95% CI)	*p*-value
Test result						
Not tested	164	7 (4.3)	1		1	
mRDT negative	2558	244 (9.5)	2.32 (0.97–5.54)	0.151	2.50 (0.84–7.44)	0.144
mRDT positive	93	12 (12.9)	2.61 (0.90–7.63)		3.70 (0.94–14.56)	
Age group						
< 1.0	820	73 (8.9)	1		1	
1.0–2.9	1169	119 (10.2)	1.05 (0.76–1.44)		1.13 (0.80–1.61)	
3.0–4.9	788	69 (8.8)	0.95 (0.66–1.37)	0.951	1.04 (0.70–1.55)	0.768
5.0–15.0	10	0 (0.0)	n/a		1.00 (0.70–1.55)	
Gender						
Male	1463	134 (9.2)	1		1	
Female	1330	126 (9.5)	1.07 (0.82–1.40)	0.628	1.04 (0.78–1.39)	0.778
Slept under a net the previous night						
No	267	27 (10.1)	1		1	
Yes	2500	232 (9.3)	0.87 (0.54–1.41)	0.579	1.04 (0.61–1.74)	0.897
Resident in the same village as a CHW						
No	317	23 (7.3)	1		1	
Yes	2490	239 (9.6)	1.43 (0.89–2.30)	0.137	1.17 (0.71–1.92)	0.536
Time of presentation to CHW after the onset of symptoms						
> 24 h	408	54 (13.2)	1		1	
Within 24 h	2321	202 (8.7)	0.68 (0.48–0.96)	0.030	0.50 (0.37–0.69)	< 0.001
Type of referral						
Severe referral	1013	152 (15.0)	1		1	
Non-severe referral	1607	111 (6.9)	0.47 (0.35–0.64)	< 0.001	0.63 (0.44–0.90)	0.012
ACT prescription						
No ACT	2538	242 (9.5)	1		1	
ACT	82	8 (9.8)	1.01 (0.45–2.25)	0.978	1.70 (0.54–5.41)	0.368
Day of visit to CHW						
Weekday	2022	211 (10.4)	1	0.002	1	
Weekend	793	52 (6.6)	0.60 (0.44–0.84)		0.63 (0.45–0.89)	0.009
Season of visit						
Dry	1139	142 (12.5)	1	< 0.001	1	
Wet	1676	121 (7.2)	0.58 (0.44–0.76)		0.56 (0.42–0.74)	< 0.001
Village distance to nearest health facility (km)						
0.0–2.4	1578	189 (12.0)	1		1	
2.5–4.9	1141	74 (6.5)	0.46 (0.25–0.85)		0.49 (0.25–0.94)	0.019
5.0–7.4	72	0 (0.0)	n/a	0.013	n/a	
7.5–8.9	0	0 (0.0)	n/a		n/a

**Table 6:** Caregiver factors associated with referral compliance in the low-transmission setting

Variables	Referrals by CHWs	Complied with referral (%)	Unadjusted odds Ratio (95% CI)	*p*-value	Adjusted odds ratio (95% CI)	*p*-value
Test result						
Not tested	352	34 (9.7)	1		1	
mRDT negative	770	75 (9.7)	0.95 (0.44–2.05)	0.735	0.50 (0.21–1.18)	0.249
mRDT positive	13	3 (23.1)	1.87 (0.30–11.70)		1.30 (0.09–18.35)	
Age group						
< 1.0	324	29 (9.0)	1		1	
1.0–2.9	483	49 (10.1)	1.02 (0.60–1.74)		1.41 (0.76–2.62)	
3.0–4.9	302	30 (9.9)	1.00 (0.55–1.82)	0.996	1.08 (0.54–2.18)	0.490
5.0–15.0	9	1 (11.1)	0.74 (0.06–9.38)		1.00 (0.54–2.18)	
Sex						
Male	585	45 (7.7)	1		1	
Female	544	64 (11.8)	1.78 (1.14–2.77)	0.011	1.88 (1.12–3.15)	0.018
Slept under a net the previous night						
No	129	15 (11.6)	1		1	
Yes	984	93 (9.5)	0.92 (0.48–1.78)	0.815	0.63 (0.29–1.36)	0.237
Resident in the same village as a CHW						
No	187	12 (6.4)	1		1	
Yes	945	100 (10.6)	1.51 (0.74–3.09)	0.254	1.94 (0.68–5.52)	0.215
Time of presentation to CHW after the onset of symptoms						
> 24 h	220	20 (9.1)	1		1	
Within 24 h	879	83 (9.4)	1.22 (0.70–2.14)	0.483	0.94 (0.53–1.66)	0.827
Type of referral						
Severe referral	421	41 (9.7)	1		1	
Non-severe referral	636	71 (11.2)	0.81 (0.50–1.31)	0.392	1.33 (0.70–2.52)	0.391
ACT prescription						
No ACT	805	86 (10.7)	1		1	
ACT	218	10 (4.6)	0.30 (0.12–0.72)	0.007	0.24 (0.09–0.65)	0.005
Day of visit to CHW						
Weekday	791	94 (11.9)	1	0.001	1	
Weekend	344	18 (5.2)	0.39 (0.22–0.69)		0.35 (0.18–0.68)	0.002
Season of visit						
Dry	419	40 (9.5)	1	0.938	1	
Wet	716	72 (10.1)	0.98 (0.63–1.54)	0.938	0.93 (0.55–1.58)	0.798
Village distance to nearest health facility (km)						
0.0–2.4	296	20 (6.8)	1		1	
2.5–4.9	395	10 (2.5)	0.37 (0.15–0.94)		0.32 (0.11–0.89)	
5.0–7.4	241	44 (18.3)	2.81 (1.30–6.07)	< 0.001	2.94 (1.19–7.24)	< 0.001
7.5–8.9	130	32 (24.6)	5.10 (2.05–12.71)		3.25 (1.15–9.21)	

In the low transmission setting, caregivers of female children were more likely to comply with referral compared to males (OR 1.88; 95%CI 1.12–3.15; *p* = 0.018) and the likelihood of compliance was 76% (OR 0.24; 95% CI 0.09–0.65; *p* = 0.005) less amongst children prescribed an ACT compared to children not prescribed an ACT (Table 6). Several of the factors associated with referral compliance in the moderate-to-high transmission setting were also associated with referral in the low transmission setting. Referral during a weekend compared to a weekday was less likely to result in compliance (OR 0.35; 95%CI 0.18–0.68; *p* = 0.002) and increasing village distance from the nearest health centre was significantly associated with referral compliance (Table 6).

A supplementary analysis of these referral forms was undertaken to examine caregivers referral compliance according to the specific signs and symptoms reported by CHWs in each transmission setting (Additional file 2: Table S1, Additional file 3: Table S2). Caregivers in both settings nearly always complied with referral when either a non-severe or a severe sign or symptom was reported. The sole exception to this pattern was when a measured temperature of > 37 °C and mRDT negative test results were recorded as the reason for referral (Additional file 2: Table S1, Additional file 3: Table S2).

### Health centre management of referred cases

The case management decisions taken by health workers at health centres when caregivers complied with CHW referral advice were reported on referral forms and these were examined to describe the final child diagnoses. In both transmission settings, most children were diagnosed and treated the same day in health centre outpatient departments, however a few children were admitted (moderate-to-high transmission setting: 18/208, 36 missing data, low-transmission setting: 2/89, 23 missing data) and one child in the moderate-to-high transmission setting was referred from a health centre to a hospital. No deaths were reported in either setting. The diagnoses made by health workers in both transmission settings were broadly similar. Children tested with a mRDT by CHWs in the moderate-to-high transmission setting were frequently diagnosed with respiratory tract infections (RTI) (32.9%), helminths (24.3%), malaria (18.1%) and diarrhoea (10.7%), and in the low transmission setting, RTI (66.7%), malaria (9.7%) and diarrhoea (8.3%) were the most frequent diagnoses (Additional file 4: Table S3, Additional file 5: Table S4). The frequency and types of diagnoses reported by health workers among mRDT tested children were predominantly due to children who were mRDT negative by CHW and compliant with the referral. In the moderate-to-high transmission setting, 21 different diagnoses were reported amongst mRDT negative children whilst only 3 diagnoses were reported for mRDT positive children. There was consistent agreement between the diagnoses made by CHW and health workers, in the moderate-to-high transmission setting 156/169 found by CHW to be mRDT negative were also found to mRDT negative by health workers. However, 8/169 (4.7%) children found to mRDT negative by health workers were given a malaria diagnosis, suggesting health workers did not agree with the negative test result. The agreement was also consistent in the low transmission setting, 23/27 mRDT negative children were also found negative by health workers whilst, 4/27 (14.8%) were mRDT positive and given a malaria diagnosis.

## Discussion

In these studies, less than 10% of caregivers in rural Uganda adhered to the referral advice given by CHW trained to identify referral signs and symptoms in children under-5 in two different malaria transmission settings. There was a trend suggesting that testing for malaria with mRDTs in the moderate-to-high transmission setting increased caregivers compliance to referral advice compared with a presumptive diagnosis. However, there was no association between compliance and the mRDT result or ACT treatment in the multivariable analyses. The study also found compliance was greater when children presented with severe referral signs and symptoms compared with non-severe signs and symptoms in the moderate-to-transmission setting. Whilst there was some evidence of an association between mRDT testing and compliance in the moderate-to-high transmission setting, there was no association in the low transmission setting. Also, there was evidence that caregivers of children who were not treated with an ACT were more likely to comply with referral advice compared to caregivers of children treated by the CHW. The difference in compliance according to the severity of signs or symptoms might suggest that caregivers also applied their own judgement in deciding which symptoms required higher level management at health centres. Despite the poor overall compliance in both settings, there was evidence to suggest that amongst caregivers who complied with referral advice, many did so within 1 day of being referred.

CHWs in the study were also trained to give pre-referral rectal artesunate to children presenting with signs and symptoms of severe malaria. However, nearly all children treated with rectal artesunate failed to comply with the referral advice. This might be explained by an immediate improvement of signs and symptoms after administering pre-referral treatment and caregivers perceiving there is no longer a necessity to seek treatment. This is particularly concerning, because the failure to seek further curative treatment after pre-referral artesunate may lead to severe disease or a recrudescence of malaria because approximately one-third of children can still be parasitemic after receiving rectal-artesunate [29, 30]. To improve compliance to referral advice amongst this high-risk group, training materials of CHW should emphasise more strongly that rectal artesunate is not a full curative treatment for malaria and that further care should be sought from health centres.

In both transmission settings, caregivers were less likely to comply when referred during the weekend compared to on weekdays. In the moderate-to-high transmission setting children living in villages further away from health centres were less likely to comply with referral advice, whilst in the low transmission setting caregivers living further away from a health facility were more likely to comply. This counterintuitive finding in the low transmission setting may partly be explained by a health insurance scheme run by a private hospital in the low-transmission setting, this may have facilitated timely use of healthcare services compared to the moderate to high transmission setting that lacked an insurance scheme. There may also have been differences in caregivers’ perceptions of the seriousness of a malaria diagnosis, in this high-altitude epidemic-prone setting where acquire immunity to malaria can be lower and malaria can be deadlier compared to an endemic setting.

In the moderate-to-high setting caregivers who visited CHWs within 24 h of symptoms starting were less likely to comply with referral advice compared to children who visited CHW more than 24 h and children referred during the wet season were less likely to comply compared with children referred during the dry season. By contrast, in the low-transmission setting, there was no association between compliance and the time of fever symptom onset or season. There was a trend that female children were more likely to comply with referral compared to males and compliance was unlikely when children were prescribed ACT compared to when an ACT was not prescribed. This suggests that having received a malaria treatment from the CHW, caregivers decided not to seek further care from health centres, despite being referred by CHW for other symptoms. This may also be a concern because ACTs are intended to treat uncomplicated cases of malaria and management should be sought for children who had other referral signs and symptoms.

Reviewing and linking referral forms completed by CHW and health centre workers, was advantageous as it enabled an assessment of referral compliance on a large sample of referred children and allowed an exploration of several geographical and temporal factors likely to be associated with compliance, such as age, sex, distance and seasonality. However, there are some disadvantages to this method of record linkage. For example, the CHWs reported that caregivers sometimes refused to accept referral forms upon being referred by CHW, indicating that the CHW may not always have issued a referral form. It is also possible that caregivers might leave the forms at home, or that health workers misplaced the forms of children taken to a health centre. In any of these situations, the absence of referral forms at the health centre would be interpreted as the caregiver failing to comply with the referral advice, which may underestimate actual compliance, for example if caregivers had visited without a form. Secondly, routinely available data from the treatment recording forms were used to examine factors likely to be associated with referral. However, these forms did not capture data on several other potentially relevant factors such as socio-economic and educational status of caregivers that may also be associated with compliance. Due to logistical constraints it was not possible to conduct follow-up household visits with all referrals which could have provided more information related to referral compliance, such as education, socio-economic status and attitudes and perceptions towards medicines [16]. A related cost-effectiveness study reported the household costs for caregivers complying with the referral, which might suggest increased household costs for referral may be a consideration when deciding to comply with referral [31]. Finally, this study did not follow-up caregivers who did not comply with the referral advice and therefore the health outcomes of these children were not assessed. Further research is required to understand the reasons for poor compliance and the health outcomes of children who do not comply with referral. Finally, this study did not follow-up caregivers who did not comply with the referral advice and therefore the health outcomes of these children were not assessed. Neither did it seek to examine whether CHW’s decision to refer caregivers was correct. It is possible that CHWs referred children who did not require referral and the caregivers’ non-compliance to referral advice had no negative consequence in terms of health outcomes. Further research is required to understand the reasons for poor compliance and the health outcomes of children who do not comply with referral. A qualitative investigation of attitudes towards referral from the perspectives of the caregiver and CHW may help to further understand referral and care seeking behaviours and inform future intervention strategies.

Despite the challenges of tracking referrals in this analysis, the results are consistent with other previous studies investigating referral compliance from community settings which also report low compliance. Studies in Sierra Leone and Zambia with CHW-managed malaria with mRDTs and ACTs found caregivers’ compliance ranged from 2% to 46% respectively [13, 32]. More recent iCCM referral studies also found suboptimal compliance ranging from 30% to 46% with both iCCM studies also identifying distance to the health centre and household costs of referral to be barriers to access [15, 16].

The findings from these studies in Uganda and elsewhere show that community based referral systems operate less than optimally at each stage of the referral process. In the first stage, CHW often do not refer children with referral signs and symptoms to the nearest health centre [23, 33, 34]. In the second stage, caregivers often fail to comply with CHW referral advice and do not seek care from health centres [13–16]. The combined effect of both the failure of CHWs to refer eligible children and the poor compliance to referral advice by caregivers risks undermining the full effectiveness of community based treatment programmes that aim to reduce child mortality by providing primary healthcare services closer to populations with poor access to health centres.

A functional and appropriately managed referral system is an essential component of primary healthcare yet remains poorly understood. In acknowledgement of the current evidence gaps regarding referral the international task force on iCCM has highlighted this as a global research priority [17, 35]. The evidence presented here provides some evidence that CHWs can make appropriate referrals and caregivers comply with referral advice. It also raises additional research questions requiring further investigation to better inform recommendations and guidelines for countries implementing community programmes. First, our findings indicate that referral compliance may differ depending on the sign or symptom, and referral guidelines and communication with caregivers may thus need to address both clinical priorities and local caregivers’ perceptions of the severity of symptoms. Second, better coordination and monitoring of referrals from the CHW to health centres is required to track caregivers’ compliance and the health outcomes of children. Second, CHWs play an important role in advising caregivers on further treatment options. CHW training could include counselling caregivers about the importance of complying with referral and discussing alternative solutions to overcome barriers to seeking further care. This is particularly important for caregivers who fail to comply with referral advice after their child receives pre-referral rectal artesunate. Third, further research is needed on barriers and enablers of caregiver’s compliance with referral advice. Fourth, better coordination and monitoring of referrals from the CHW to health centres is required to track caregivers’ compliance and the health outcomes of children. Finally, health centres should be equipped and managed to receive referred cases effectively. For example, priority could be given to referral cases upon arrival allowing them to bypass waiting in outpatient departments. Finally, health centres should be equipped and managed to receive referred cases effectively. For example, priority could be given to referral cases upon arrival allowing them to bypass waiting in outpatient departments.

The referral challenges faced by community based programmes may be similar to the challenges to access and utilisation of health services faced by other health programmes. Interventions developed to improve attendance at health centres for pregnant women and utilisation of antenatal care and new-born health, could also be relevant to iCCM programmes to improve health seeking behaviour amongst referred caregivers [36]. Findings from this field may also be adapted to iCCM programmes to improve health seeking behaviour amongst referred caregivers. For example, interventions that involve regular home visits by CHWs to prepare pregnant women for birth and immediate new-born care could also be adapted to iCCM programmes where CHWs regularly follow-up referred caregivers to encourage compliance and offer further support and counselling on the importance of referral. An important barrier to accessing public health centres are the household costs associated with seeking care [37]. Financial incentives such as conditional cash transfers (CCTs) aim to offset some of the household financial burden associated with health seeking and CCT interventions have shown to increase the use of health centres for ANC services in Latin American and South East Asian countries [38]. Further research could explore whether particular models of CCTs within iCCM programmes could also improve caregiver’s compliance with referral advice.

## Conclusion

In two randomised controlled trials that evaluated the effectiveness of training CHWs to diagnose malaria using mRDT in Uganda, the majority of caregivers of children with a febrile illness did not comply with the referral advice given by CHW. This is particularly concerning for children with signs of severe disease, including children with severe malaria who received pre-referral treatment with rectal artesunate. Such children are beyond the capacity of CHW and lack of follow up treatment increases the risk of recrudescence, health complications and possible death. The findings also identified multiple geographical and temporal treatment seeking barriers associated with poor compliance. As countries in sub-Saharan Africa continue the scale-up of community based programmes, interventions to diminished barriers to accessing first level referral services are needed to ensure the continuum of care from the community to the health centre.

Over the past decade Uganda and 33 SSA countries have implemented community case management programmes as part of national healthcare strategies and despite the considerable literature on addressing the bottlenecks to scaling-up programmes there has been relatively limited evidence on strengthening referral systems as part of community programmes. The referral system is an important part of primary healthcare to improve access to appropriate care for children with conditions that cannot be managed by CHW. However, unless the referral barriers to comply with referral advice are overcome the full potential of community based programmes may not be achieved.

## Additional files

Additional file 1: **Figure S1.** Description of data: Treatment flow chart for CHWs a) Intervention arm, b) Control arm. (PDF 494 kb)

Additional file 2: **Table S1.** Description of data: Referral compliance according to child’s signs and symptoms recorded on referral forms in the moderate-to-high transmission setting. (PDF 455 kb)

Additional file 3: **Table S2.** Description of data: Referral compliance according to child’s signs and symptoms recorded on referral forms in the low transmission setting. (PDF 455 kb)

Additional file 4: **Table S3.** Description of data: Diagnoses of children made at health centres for children complying with CHW referral advice in the moderate-to-high transmission setting. (PDF 574 kb)

Additional file 5: **Table S4.** Description of data: Diagnoses of children made at health centres for children complying with CHW referral advice in the low transmission setting. (PDF 579 kb)

## Abbreviations

95% CI: 95% confidence intervals
ACT: Artemisinin-based combination therapy
AOR: Adjusted odds ratio
CHW: Community health worker
HCII: Health centre II
HCIII: Health centre III
iCCM: Integrated community case management
ITN: insecticide treated net
mRDTs: Malaria rapid diagnostic tests
OR: Odds ratio
