# Supplementary figures and images for: Caregivers’ compliance with referral advice: evidence from two studies introducing mRDTs into community case management of malaria in Uganda
Source: BMC Health Serv Res. 2018 May 2;18:317. doi: 10.1186/s12913-018-3124-8 (PMC5932808; doi:10.1186/s12913-018-3124-8)

Fig S1: Treatment flow chart for CHWs a) Intervention arm, b) Control arm

a)

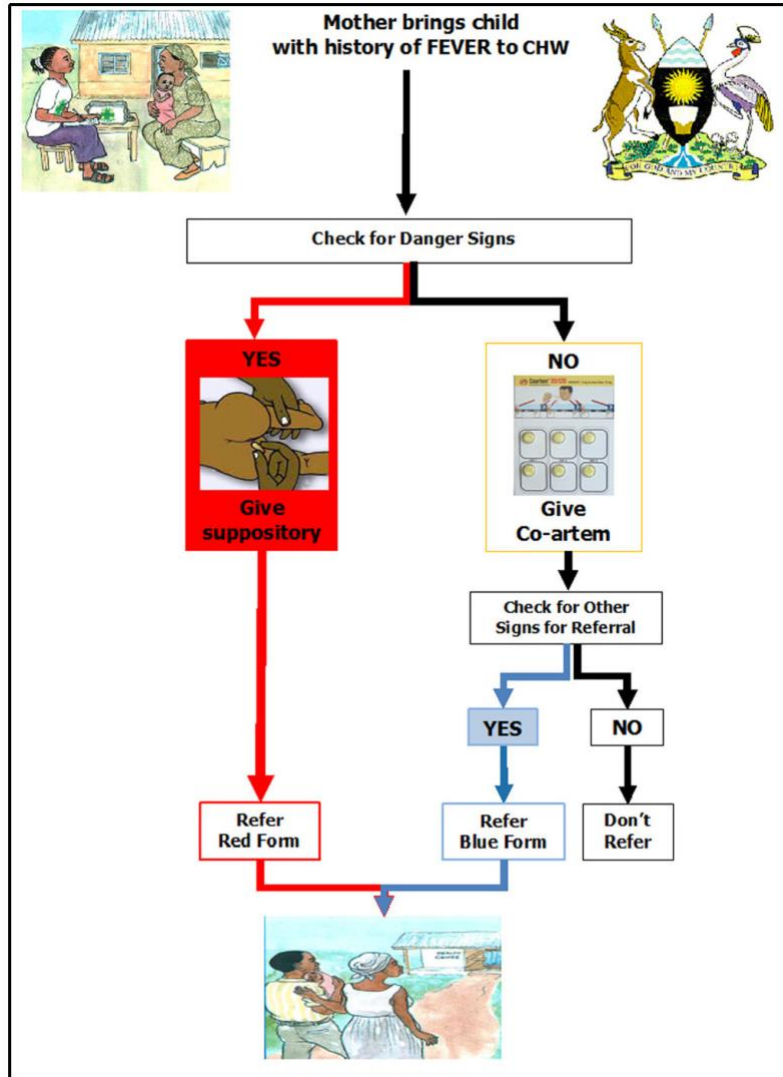

b)

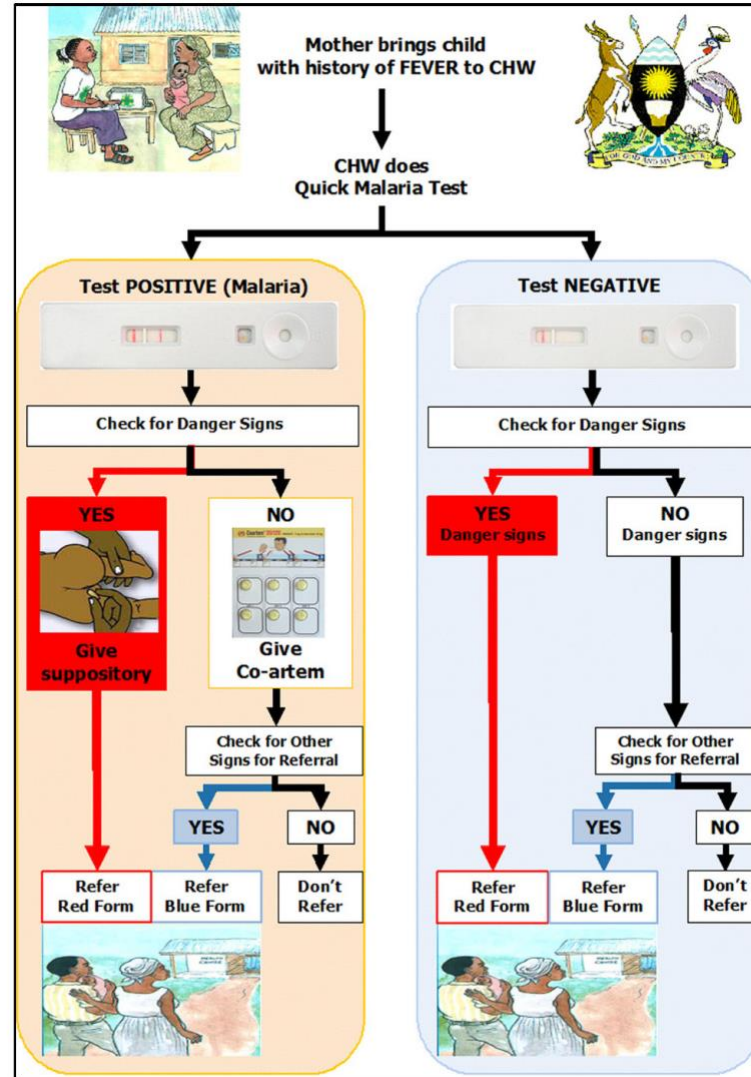

Supplement: Supplementary file 1 — Figure S1. Description of data: Treatment flow chart for CHWs a) Intervention arm, b) Control arm. (PDF 494 kb) [file 12913_2018_3124_MOESM1_ESM.pdf]
