# Supplementary material for: Caregivers’ compliance with referral advice: evidence from two studies introducing mRDTs into community case management of malaria in Uganda
Source: BMC Health Serv Res. 2018 May 2;18:317. doi: 10.1186/s12913-018-3124-8 (PMC5932808; doi:10.1186/s12913-018-3124-8)
Supplement: Supplementary file 2 — Table S1. Description of data: Referral compliance according to child’s signs and symptoms recorded on referral forms in the moderate-to-high transmission setting. (PDF 455 kb) [file 12913_2018_3124_MOESM2_ESM.pdf]

Table S1: Referral compliance according to child's signs and symptoms recorded on referral forms in the moderate-to-high transmission setting

	Total[a]	Complied with referral<br>Frequency (%)	Did not comply with referral<br>Frequency (%)
Severe signs and symptoms for referral			
Illness in child below 2 months	2	2 (100.0)	0
Convulsions or fits now or within the past 2 days	22	21 (95.5)	1
Coma / Loss of consciousness	6	6 (100.0)	0
Patient is confused or very sleepy - cannot be woken	5	5 (100.0)	0
Extreme weakness unable to stand or sit without support	12	12 (100.0)	0
Very Hot with temperature of 38.5°C or more	81	80 (98.8)	1
Very Cold with temperature of 35.0°C or less	10	10 (100.0)	0
Vomiting everything - cannot keep down food or drink	36	36 (100.0)	0
Not able to drink or breastfeed	53	52 (98.1)	1
Severe anaemia very pale palms, fingernails, eyelids	19	19 (100.0)	0
Yellow eyes	9	9 (100.0)	0
Difficulty in breathing	73	72 (98.6)	1
Severe dehydration	7	7 (100.0)	0
Other severe signs and symptoms[b]	34	34 (100.0)	0
**Total number of severe signs and symptoms reported**	369	365	338
Total number of severe referral forms	151	150 (99.3)	1 (0.7)
Mean number of signs and symptoms reported per severe referral form	2.4	2.4	338
Non-severe signs and symptoms for referral			
Fever in babies less than 4 months old	0	-	-
Fever that has lasted more than 7 days	16	16 (100.0)	0
Fever with measured temperature of > 37.0°C and mRDT Negative	214	0 (0.0)	214
Vomiting and diarrhoea	32	31 (96.9)	1
Blood in faeces or urine	2	2 (100.0)	0
Pain when passing urine, or frequent urination	8	8 (100.0)	0
Wounds or Burns	8	8 (100.0)	0
Skin abscess	3	3 (100.0)	0
Painful swellings or lumps in the skin	3	3 (100.0)	0
Ear infection (runny ear or child pulling at ear)	1	1 (100.0)	0
Sticky or red eyes	16	16 (100.0)	0
Other non-severe signs and symptoms[c]	35	35 (100.0)	0
**Total number of non-severe signs and symptoms reported**	338	123	215
Total number of non-severe referrals forms	313	98 (31.3)	215 (68.7)
Mean number of signs and symptoms reported per referral	1.1	1.3	1.0

a The number of reported signs and symptom is lower than the referrals reported in the main tables. This is because not all referrals reported on the treatment recording forms were accompanied with the separate referral forms.

b Other severe signs and symptoms included: complied with referral; cough and flu (20), diarrhoea (5), eye problems (2), burns (1), painful ear (1), unable to eat or drink (2), yellow skin (1), dysentery (1), severe headaches (1)

c Other non-severe signs and symptoms for referral included: complied with referral; cough and flu (15), high temperature (17), worms (1), swollen legs and eyes (1), Unknown (1); Did not comply with previous referral; cough flu (1).
