# Supplementary material for: Caregivers’ compliance with referral advice: evidence from two studies introducing mRDTs into community case management of malaria in Uganda
Source: BMC Health Serv Res. 2018 May 2;18:317. doi: 10.1186/s12913-018-3124-8 (PMC5932808; doi:10.1186/s12913-018-3124-8)
Supplement: Supplementary file 3 — Table S2. Description of data: Referral compliance according to child’s signs and symptoms recorded on referral forms in the low transmission setting. (PDF 455 kb) [file 12913_2018_3124_MOESM3_ESM.pdf]

Table S2: Referral compliance according to child's signs and symptoms recorded on referral forms in the low transmission setting[a]

	Total[a]	Complied with referral<br>Frequency (%)	Did not comply with referral<br>Frequency (%)
Severe signs and symptoms for referral			
Illness in child below 2 months	1	1 (100.0)	0 (0.0)
Convulsions or fits now or within the past 2 days	2	2 (100.0)	0 (0.0)
Coma / Loss of consciousness	1	1 (100.0)	0 (0.0)
Patient is confused or very sleepy - cannot be woken	2	2 (100.0)	0 (0.0)
Extreme weakness unable to stand or sit without support	3	3 (100.0)	0 (0.0)
Very Hot with temperature of 38.5°C or more	32	32 (100.0)	0 (0.0)
Very Cold with temperature of 35.0°C or less	3	3 (100.0)	0 (0.0)
Vomiting everything - cannot keep down food or drink	8	8 (100.0)	0 (0.0)
Not able to drink or breastfeed	8	8 (100.0)	0 (0.0)
Severe anaemia very pale palms, fingernails, eyelids	2	2 (100.0)	0 (0.0)
Yellow eyes	1	1 (100.0)	0 (0.0)
Difficulty in breathing	22	22 (100.0)	0 (0.0)
Severe dehydration	1	1 (100.0)	0 (0.0)
Other severe signs and symptoms[b]	7	7 (100.0)	0 (0.0)
**Total number of severe signs and symptoms reported**	93	114	114
Total number of severe referral forms	41	41	0
Mean number of signs and symptoms reported per severe referral form	0.0	2.3	0.0
Non-severe signs and symptoms for referral			
Fever in babies less than 4 months old	0	-	-
Fever that has lasted more than 7 days	4	4 (100.0)	0 (0.0)
Fever with measured temperature of > 37.0°C and mRDT Negative	37	0 (0.0)	37 (100.0)
Vomiting and diarrhoea	21	21 (100.0)	0 (0.0)
Blood in faeces or urine	0	0 (0.0)	0 (0.0)
Pain when passing urine, or frequent urination	4	4 (100.0)	0 (0.0)
Wounds or Burns	2	2 (100.0)	0 (0.0)
Skin abscess	0	0 (0.0)	0 (0.0)
Painful swellings or lumps in the skin	2	2 (100.0)	0 (0.0)
Ear infection (runny ear or child pulling at ear)	0	0 (0.0)	0 (0.0)
Sticky or red eyes	1	1 (100.0)	0 (0.0)
Other non-severe signs and symptoms[c]	43	43 (100.0)	0 (0.0)
**Total number of non-severe signs and symptoms reported**	114	77	37
Total number of non-severe referrals forms	98	61 (62.2)	37 (37.8)
Mean number of signs and symptoms reported per referral	1.2	1.3	1.0

a The number of reported signs and symptom is lower than the referrals reported in the main tables. This is because not all referrals reported on the treatment recording forms were accompanied with the separate referral forms.
b Other severe signs and symptoms included: complied with referral; cough and flu (20), diarrhoea (5), eye problems (2), burns (1), painful ear (1), unable to eat or drink (2), yellow skin (1), dysentery (1), severe headaches (1)
c Other non-severe signs and symptoms for referral included: complied with referral; cough and flu (15), high temperature (17), worms (1), swollen legs and eyes (1), Unknown (1); Did not comply with previous referral; cough flu (1).
