# Supplementary material for: Caregivers’ compliance with referral advice: evidence from two studies introducing mRDTs into community case management of malaria in Uganda
Source: BMC Health Serv Res. 2018 May 2;18:317. doi: 10.1186/s12913-018-3124-8 (PMC5932808; doi:10.1186/s12913-018-3124-8)
Supplement: Supplementary file 4 — Table S3. Description of data: Diagnoses of children made at health centres for children complying with CHW referral advice in the moderate-to-high transmission setting. (PDF 574 kb) [file 12913_2018_3124_MOESM4_ESM.pdf]

Table S3: Diagnoses of children made at health centres for children complying with CHW referral advice in the moderate-to-high transmission setting

Diagnoses made by health centre staff	Total Frequency (%)[a]	Not tested with mRDT at health centre Frequency (%)	Tested with mRDT at health centre Frequency (%)	*mRDT test result: Positive Frequency (%)*	*mRDT test result: Negative Frequency (%)*
Abscess	1 (0.0)	0 (0.0)	1 (100.0)	*0 (0.0)*	*1 (100.0)*
Anaemia	1 (0.0)	0 (0.0)	1 (100.0)	*0 (0.0)*	*1 (100.0)*
Bacterial conjunctivitis	1 (0.0)	0 (0.0)	1 (100.0)	*0 (0.0)*	*1 (100.0)*
Burns	2 (0.0)	0 (0.0)	2 (100.0)	*0 (0.0)*	*2 (100.0)*
Diarrhoea	28 (0.1)	2 (7.1)	26 (92.9)	*0 (0.0)*	*26 (100.0)*
Ear wound	-	-	-	-	-
Epilepsy	1 (0.0)	0 (0.0)	1 (100.0)	*0 (0.0)*	*1 (100.0)*
Epistaxis	-	-	-	-	-
Eye infection	2 (0.0)	1 (50)	1 (50)	*0 (0.0)*	*1 (100.0)*
Flu	80 (0.3)	0 (0.0)	80 (100.0)	*2 (2.5)*	*78 (97.5)*
Gastroenteritis	1 (0.0)	0 (0.0)	1 (100.0)	*0 (0.0)*	*1 (100.0)*
Helminths	60 (0.2)	1 (1.7)	59 (98.3)	*0 (0.0)*	*59 (100.0)*
Malaria	45 (0.2)	1 (2.2)	44 (97.8)	*6 (13.6)*	*38 (86.4)*
Mumps	3 (0.0)	1 (33.3)	2 (66.7)	*0 (0.0)*	*2 (100.0)*
Oral candidiasis	0 (0.0)	-	-	-	-
Otitis media	1 (0.0)	0 (0.0)	1 (100.0)	*0 (0.0)*	*1 (100.0)*
Pneumonia	11 (0.0)	0 (0.0)	11 (100.0)	*3 (27.3)*	*8 (72.7)*
Scalp infection	0 (0.0)	-	-	-	-
Skin infection	5 (0.0)	0 (0.0)	5 (100.0)	*0 (0.0)*	*5 (100.0)*
Toe wound	1 (0.0)	0 (0.0)	1 (100.0)	*0 (0.0)*	*1 (100.0)*
Trachoma	2 (0.0)	0 (0.0)	2 (100.0)	*0 (0.0)*	*2 (100.0)*
Trauma	1 (0.0)	0 (0.0)	1 (100.0)	*0 (0.0)*	*1 (100.0)*
Ulcer	1 (0.0)	0 (0.0)	1 (100.0)	*0 (0.0)*	*1 (100.0)*
Urinary tract infection	1 (0.0)	0 (0.0)	1 (100.0)	*0 (0.0)*	*1 (100.0)*
Vomiting	1 (0.0)	0 (0.0)	1 (100.0)	*0 (0.0)*	*1 (100.0)*
**Total diagnoses**	**249**	**6 (2.4)**	**243 (97.6)**	**11 (4.5)**	**232 (95.5)**

a Column percentages are reported for the totals.
