# Supplementary material for: Caregivers’ compliance with referral advice: evidence from two studies introducing mRDTs into community case management of malaria in Uganda
Source: BMC Health Serv Res. 2018 May 2;18:317. doi: 10.1186/s12913-018-3124-8 (PMC5932808; doi:10.1186/s12913-018-3124-8)
Supplement: Supplementary file 5 — Table S4. Description of data: Diagnoses of children made at health centres for children complying with CHW referral advice in the low transmission setting. (PDF 579 kb) [file 12913_2018_3124_MOESM5_ESM.pdf]

Table S4: Diagnoses of children made at health centres for children complying with CHW referral advice in the low transmission setting

Diagnoses made by health centre staff	Total Frequency (%)[a]	Not tested with mRDT at health centre Frequency (%)	Tested with mRDT at health centre Frequency (%)	*mRDT test result: Positive Frequency (%)*	*mRDT test result: Negative Frequency (%)*
Abscess	(0.0)	-	-	-	-
Anaemia	(0.0)	-	-	-	-
Bacterial conjunctivitis	(0.0)	-	-	-	-
Burns	(0.0)	-	-	-	-
Diarrhoea	10 (10.0)	4 (40.0)	6 (60.0)	*0 (0.0)*	*6 (100.0)*
Ear wound	1 (1.0)	0 (0.0)	1 (100.0)	*0 (0.0)*	*1 (100.0)*
Epilepsy	(0.0)	-	-	-	-
Epistaxis	1 (1.0)	0 (0.0)	1 (100.0)	*0 (0.0)*	*1 (100.0)*
Eye infection	(0.0)	-	-	-	-
Flu	63 (63.0)	15 (23.8)	48 (76.2)	*1 (2.1)*	*47 (97.9)*
Gastroenteritis	1 (1.0)	0 (0.0)	1 (100.0)	*0 (0.0)*	*1 (100.0)*
Helminths	1 (1.0)	0 (0.0)	1 (100.0)	*0 (0.0)*	*1 (100.0)*
Malaria	9 (9.0)	2 (22.2)	7 (77.8)	*2 (28.6)*	*5 (71.4)*
Mumps	(0.0)	-	-	-	-
Oral candidiasis	1 (1.0)	1 (100.0)	0 (0.0)	-	-
Otitis media	1 (1.0)	0 (0.0)	1 (100.0)	*0 (0.0)*	*1 (100.0)*
Pneumonia	8 (8.0)	2 (25.0)	6 (75.0)	*0 (0.0)*	*6 (100.0)*
Scalp infection	(0.0)	-	-	-	-
Skin infection	1 (1.0)	1 (100.0)	0 (0.0)	-	-
Toe wound	(0.0)	-	-	-	-
Trachoma	(0.0)	-	-	-	-
Trauma	(0.0)	-	-	-	-
Ulcer	(0.0)	-	-	-	-
Urinary tract infection	3 (3.0)	3 (100.0)	0 (0.0)	-	-
Vomiting	(0.0)	-	-	-	-
**Total diagnoses**	**100**	**28 (28.0)**	**72 (72.0)**	***3 (4.2)***	***69 (95.8)***

a Column percentages are reported for the totals.
